# SDTCP: Towards Datacenter TCP Congestion Control with SDN for IoT Applications

**DOI:** 10.3390/s17010109

**Published:** 2017-01-08

**Authors:** Yifei Lu, Zhen Ling, Shuhong Zhu, Ling Tang

**Affiliations:** 1 School of Computer Science and Engineering, Nanjing University of Science and Technology, Nanjing 210094, China; stuart.chu@hotmail.com (S.Z.); ling.tang@njust.edu.cn (L.T.); 2 School of Computer Science and Engineering, Southeast University, Nanjing 211189, China; zhenling@seu.edu.cn; 3 Key Laboratory of Computer Network and Information Integration, Southeast University, Ministry of Education, Nanjing 211189, China

**Keywords:** data center networks, IoT, incast, congestion control, SDN

## Abstract

The Internet of Things (IoT) has gained popularity in recent years. Today’s IoT applications are now increasingly deployed in cloud platforms to perform Big Data analytics. In cloud data center networks (DCN), TCP incast usually happens when multiple senders simultaneously communicate with a single receiver. However, when TCP incast happens, DCN may suffer from both throughput collapse for TCP burst flows and temporary starvation for TCP background flows. In this paper, we propose a software defined network (SDN)-based TCP congestion control mechanism, referred to as SDTCP, to leverage the features, e.g., centralized control methods and the global view of the network, in order to solve the TCP incast problems. When we detect network congestion on an OpenFlow switch, our controller can select the background flows and reduce their bandwidth by adjusting the advertised window of TCP ACK packets of the corresponding background flows so as to reserve more bandwidth for burst flows. SDTCP is transparent to the end systems and can accurately decelerate the rate of background flows by leveraging the global view of the network gained via SDN. The experiments demonstrate that our SDTCP can provide high tolerance for burst flows and achieve better flow completion time for short flows. Therefore, SDTCP is an effective and scalable solution for the TCP incast problem.

## 1. Introduction

The Internet of Things (IoT) is getting popular all over the world. Currently, IoT devices are used to sense/collect data and send the data to cloud s for further processing and analysis, such as Big Data analytics on real-time sensor streams [1]. The data center networks (DCN), which are the primary infrastructures for the delivery of cloud services, play an important role in data transmission to satisfy the diverse IoT applications. In today’s DCN, TCP has been used as the de facto transport layer protocol to ensure reliable data delivery. However, the unique workloads scale and environments of the data center violate the WAN (wide area network) assumptions on which TCP was originally designed. A well-known reported open problem, i.e., TCP incast [2,3], was initially identified in distributed storage clusters [2] and has nowadays become a practical issue in DCN.

**Figure 1:**
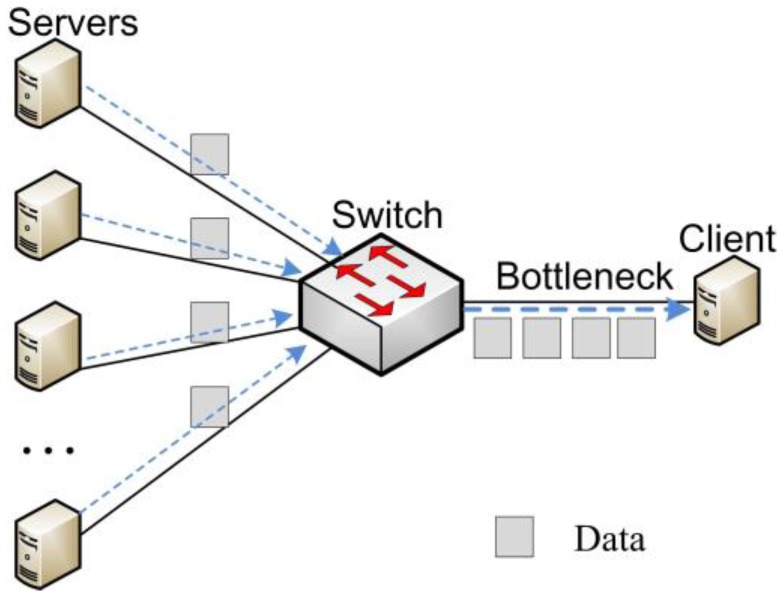
TCP incast scenario.

TCP incast occurs in synchronized many-to-one communication patterns and results in gross under-utilization of link capacity. Figure 1 shows a typical TCP incast scenario used by many studies [4,5,6]. The flows in DCN [7,8] can be classified into two groups: background flows and burst flows. The background flows are composed of large data flows that require high throughput and delay-insensitive, while burst flows consists of short control and web search flows that require low delay [9]. TCP incast would severely degrade application performance especially for those burst and delay-sensitive applications such as MapReduce [10], Dryad [11], and large-scale partition/aggregate web applications [3,12].

Previous solutions for TCP incast focused on either reducing the waiting time for packet loss recovery through faster retransmissions [13], or controlling switch buffer occupation to avoid overflow using ECN (explicit congestion notification). However, these solutions not only need to modify the TCP stack at end systems, but also ignore the characteristics of distinct flows in DCN. They do not distinguish between burst flows and background flows. Therefore, it still suffers from poor performance under bursts of burst flows due to its equitable treatment of all network flows.

In this paper, we propose a novel software defined network [14] (SDN)-based TCP congestion control mechanism, referred to as SDTCP, so as to accommodate more burst flows, achieve fast flow transmission, and improve the overall network performance and utilization. To mitigate the TCP incast problem, we carefully estimate and reduce the bandwidth of background flows in order to reserve sufficient bandwidth for burst flows. To this end, we first design a network congestion discovery mechanism by assessing queue length over an OpenFlow-enabled switch (OF-switch). Once network congestion is discovered, our OF-switch will trigger a congestion notification message to our SDN controller. Subsequently, we exploit the controller to differentiate the background flows from burst flows and estimate their available bandwidth. Then we use our customized OpenFlow protocol to send a notification message to the OF-switch. Upon receiving the notification, our OF-switch can deliberately manipulate the advertised window of TCP ACK packets of the corresponding background flows so as to effectively degrade the bandwidth of the background flows. An early version of SDTCP [15] can mitigate the TCP incast problem with a simple congestion control mechanism, while the new SDTCP introduced in this paper can optimize bandwidth assignment using a fine-grained method. To the best of our knowledge, SDTCP is the first SDN-based congestion control protocol and a new variation of TCP.

The main contributions of this paper can be summarized as follows.

(1) A SDN-based TCP congestion control mechanism is proposed in this paper and a fine-grained congestion trigger and congestion avoidance method are designed by using global view of network. SDTCP is a centralized approach that does not revise the legacy TCP stack. Thus, it is transparent to the end systems and easy to deploy. Our experimental results show that SDTCP is effective in improving the performance of burst flows, e.g., reducing transmission time, increasing the number of concurrent flows, and mitigating TCP incast collapse in DCN.
(2) By carefully deploying the location of our controller, our extensive analysis results show that the control delay between OF-switch and controller can be virtually ignored. Additionally, theoretical analysis demonstrates that flow completion time of our SDTCP is less than that of other methods and SDTCP can satisfy weighted proportional fairness.
(3) We implement SDTCP by revising the OpenFlow protocol. During TCP connection establishment, we can generate a global-view flow table (GVT) that includes all of the TCP flows’ information in the network. GVT consists of a background flows table (BGT) and a burst flows table (BRT). Then we classify the background flows and burst flows into BGT and BRT, respectively, according to different flow traffic characteristics. Furthermore, we extend the standard OpenFlow protocol to support the congestion notification message, TCP ACK flag match function, and ACK advertised window regulation action.

The rest of the paper is organized as follows: in Section 2, we review related works; in Section 3, we present the detailed design and implementation of SDTCP mechanism; we analyze the features of SDTCP in Section 4; experimental results are shown in Section 5; and, finally, we conclude this paper in Section 6.

## 2. Related Works

After the TCP incast problem was proposed by Nagle et al. in [2], plenty of existing works have been proposed to address this issue. In this section, we summarize the most relevant works. Existing solutions for the TCP incast problem can be categorized into four groups: window-based solutions, recovery-based solutions, application-based solutions, and SDN-based solutions.

### 2.1. Window-Based Solutions

The window-based solutions, e.g., DCTCP (Data Center TCP) [3] and ICTCP (Incast congestion Control for TCP) [16], control inflight traffic by adjusting the congestion or advertised window in order to evade overfilling the switch buffer. DCTCP [3] aims to ensure low latency for short flows and good utilization for long flows by reducing switch buffer occupation while minimizing buffer oscillation. DCTCP provides a congestion control scheme that utilizes the ECN feedback from congested switches to help the sender adjust the congestion window based on the network congestion. ICTCP [16] adaptively adjusts the advertised window on the receiver side to throttle aggregate throughput. ICTCP estimates available bandwidth and per-flow throughput at the receiver side every two RTTs (Round Trip Time). It only increases the advertised window when there is enough available bandwidth and the difference of measured throughput and expected throughput is small. However, ICTCP fails to work well if the bottleneck is not the link that connects to the receiver. Zhang et al. [17] propose a new transport protocol which provides fair bandwidth sharing by allocating a switch buffer (SAB) to each flow equally. SAB rarely loses packets, since SAB allocates the advertised window (*awnd*) to each flow even when *awnd* is less than one MSS (maximum segment size). SAB is similar to our SDTCP. However, SAB needs modifications at the sender, receiver, and switch side. Furthermore, SAB would consume more system resources according to modify every packet passing through the switch.

While the previous works are deadline-agnostic and focus on fair-share bandwidth allocation among flows, D^3^ [12], a deadline-driven delivery control protocol, uses explicit rate control to apportion bandwidth according to flow deadlines. Given a flow’s size and deadline, source hosts request desired rates to switches. The switches assign and reserve allocated rates for the flows. Hwang et al. [18] propose a new deadline and incast-aware TCP, called DIATCP. The key idea of DIATCP is that the aggregator can effectively obtain rich information, e.g., the bottleneck link bandwidth and the workers’ flow information including data sizes and deadlines. As a result, DIATCP operates at the aggregator to controls the peers’ sending rate directly to avoid the incast congestion. DIATCP does not require any support from the network switches and can be easily implemented and deployed at the aggregator. However, DIATCP is only suitable for a special application, like Hadoop.

### 2.2. Recovery-Based Solutions

Unlike window-based solutions, recovery-based solutions address TCP incast problem through a fast retransmission mechanism when packet loss happens. In [4], several trials have been made to avoid TCP retransmission timeout (RTO), such as reducing the duplicate ACK threshold of entering fast retransmission (FR) from three to one, disabling the slow start phase, and trying different TCP versions. Vasudevan et al. [13] suggest reducing RTO to alleviate the goodput decline and use high-resolution timers to enable microsecond-granularity TCP timeouts. In this way, TCP can retransmit lost packets quickly without leaving the link idle for a long time. CP algorithm proposed in [19] simply drops a packet’s payload at an overloaded switch and uses a SACK-like ACK mechanism to achieve rapid and precise notification of lost packets. Zhang et al. [20] propose a simple and effective solution called guarantee important packets (GIP) in DCN. In this paper, the authors observe that two types of timeouts should be avoided for improving the throughput. First, the timeouts caused by full window losses and, second, the timeouts caused by lack of duplicate ACKs. With this regard, GIP proposes two mechanisms for eliminating the two kinds of timeouts. GIP needs to modify the TCP stack.

### 2.3. Application-Based Solutions

The main idea of application-based solutions is how to use application’s information, e.g., the number of participating severs in a synchronized data transfer, to reduce packet loss. Krevat et al. [21] suppose that application knows about the parallel transmission information, e.g., the number of servers and data size. Such knowledge could be used to avoid incast by limiting the number of servers accessed at a time, staggering data transmission from those servers, and explicitly scheduling data transfers. Podlesny et al. [5,6] explore the TCP incast throughput collapse problem in DCN from an application level perspective. The main idea of this approach is to schedule the server responses to data requests so that no packet losses occur at the bottleneck link. The above approaches do not require any changes to the TCP stack or network switches, but they need to know global information and application information, which are hard to implement and deploy.

### 2.4. SDN-Based Solutions

Recently, with the wide deployment of SDN especially in DCN, the current state of the network can be aggregated at a single or a hierarchy of controllers and subsequently be used to distribute network knowledge to the end hosts in short timescales. We might wonder whether we can use the global network view available at the controller to make faster and more accurate congestion control decisions. OpenTCP [22] is presented as a system for dynamic adaptation of TCP based on network and traffic conditions in SDN. OpenTCP is not a new variation of TCP. Instead, it complements previous efforts by making it easy to switch between different TCP variants automatically, or to tune TCP parameters based on network conditions. For instance, one can use OpenTCP to either utilize DCTCP or CUBIC in a data center environment. The decision on which protocol to use is made in advance through the congestion control policies defined by the network operator. Jouet et al. [23] propose an SDN approach to tune the TCP initial window and RTO for newly-created flows based on a network-wide view. This method allows the online tuning of these parameters in order to improve the response time for mice flows by designing a measurement-based control loop with a SDN controller as the feedback source.

The major difference of our work with previous methods is that our target is to avoid packet loss by designing a new TCP congestion avoidance mechanism, while previous methods focus on how to tune TCP parameters or choose between different TCP variants automatically. This makes our work complementary to previous work.

## 3. SDTCP Mechanism

In this section, we introduce the basic ideal of our SDTCP and explain the detailed SDTCP mechanism step-by-step.

### 3.1. Basic Idea of SDTCP

**Figure 2:**
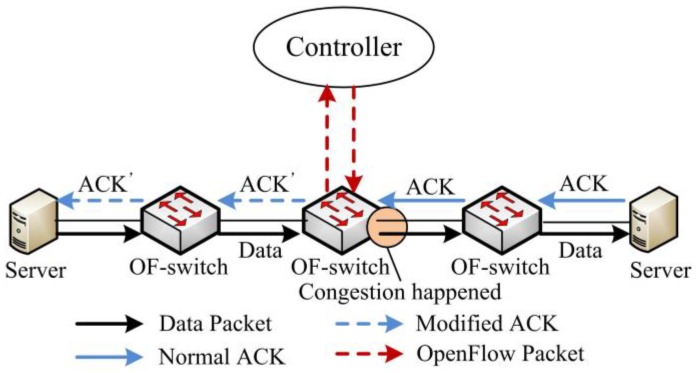
SDTCP overall architecture.

SDTCP is designed to reduce bandwidth of background flows to guarantee burst flows which are usually more urgent. To control the flow rate of the background flow, we modify the advertised window of TCP ACK packets from the receiver at our OF-switch in order to limit the flow rate of a sender as shown in Figure 2. To be specific, a TCP sender is allowed to send up to a certain number of unacknowledged bytes, referred to as send window (*swnd*), which is the minimum of sender’s congestion window (*cwnd*) and the receiver’s advertised window (*awnd*):

*swnd* = min(*cwnd*, *awnd*), (1)

In general, *cwnd* is always smaller than *awnd*, and so *swnd* is bounded by *cwnd*. Therefore, we can temporarily quench the sending rates of background flows by adjusting *awnd* in the ACK packets from the receiver at our OF-switch. Moreover, we can achieve congestion control via SDTCP without revising the legacy TCP stack at both the TCP sender and receiver.

**Figure 3:**
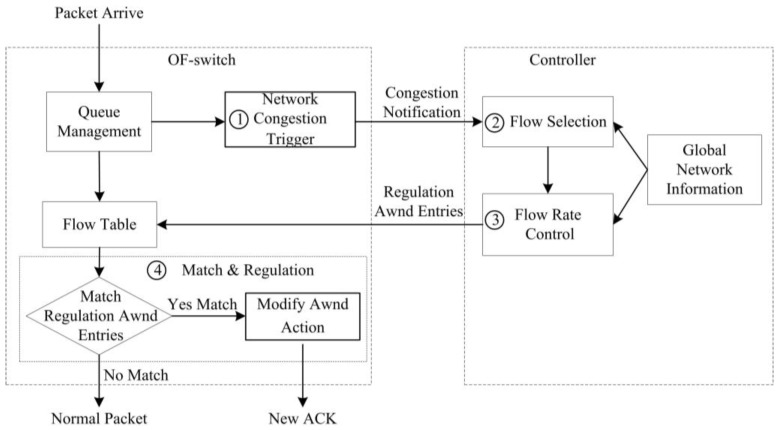
The workflow of SDTCP.

Figure 3 illustrates the workflow of our SDTCP. We briefly present the workflow as following.

**Step** **1** Network Congestion Trigger. We design a network congestion trigger module at the OF-switch to leverage the queue length to determine if the network is congested. Once network congestion is discovered, it will send a congestion notification message to our controller.
**Step** **2** Flow Selection. Our flow selection module differentiates the background flows and burst flows by leveraging all of the TCP flow information, e.g., TTL (time-to-live), flow size, and IP addresses of TCP flows, gained from OF-switches through the OpenFlow protocol. Upon receiving a congestion notification message from a congested OF-switch, our controller will select all of the background flows passing through the OF-switch.
**Step** **3** Flow Rate Control. A flow rate control module at the controller side estimates the current bandwidth of these chosen background flows and then degrades their bandwidth to the desired one. We assess our desired bandwidth in terms of the network congestion level. Then, our controller generates new flow table entries (called a regulation *awnd* entry) that is used to regulate the background flow bandwidth to our desired one and sends them to the OF-switch.
**Step** **4** Flow Match and Regulation. Once TCP ACK packets from the receiver match the regulation *awnd* entry at OF-switch, the *awnd* field of these packets will be modified to the desired one and then the packets are forwarded to the sender. After receiving these modified ACK packets, the sender will adjust *swnd* in terms of Equation (1). In this way, the sending rate can be decreased to our desired one.

We elaborate these four steps in detail below.

### 3.2. Step 1. Network Congestion Trigger

A network congestion trigger module at the OF-switch leverages the drop tail queue management to monitor the instantaneous queue length and triggers congestion notification messages when the queue length is larger than empirical thresholds. According to the congestion level, we have three different thresholds for low, modest, and high congestion. Let *L*, *M*, and *H* be the queue length thresholds corresponding to three congestion levels, respectively. Denote $q_{i}$ as the queue length. Then, we have three types of congestion notification messages:

(1) $L\leq q_{i}<M$. In this case, OF-switch triggers a congestion notification message referred to as *CN-L*.
(2) $M\leq q_{i}<H$. In this case, congestion notification message is referred to as *CN-M*.
(3) $H\leq q_{i}$. In this case, congestion notification message is referred to as *CN-H*.

After applying our congestion avoidance method presented in Step 2, Step 3, and Step 4, network congestion will be gradually alleviated. Due to the fact that 99% of the burst flows last less than 1 s [3], we consider that the network state is completely recovered if the queue length is less than L for at least 1 s. Then a congestion recovery message, referred to as a *CR* message, will be sent to our controller.

**Figure 4:**
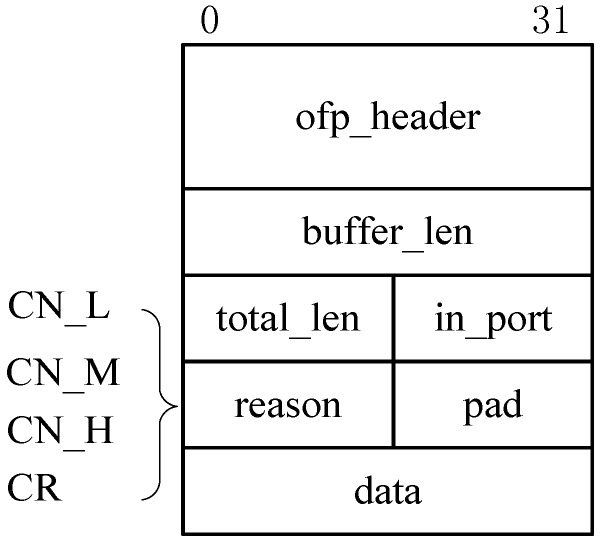
The format of the congestion notification message.

To implement this network congestion notification trigger, we customize the Openflow protocol by revising the standard Packet_In message of OpenFlow 1.3 so as to create new types of congestion and recovery notification messages. Figure 4 depicts the format of these messages that consists of message type, packet length, queue length, congested port information, etc. In particular, the *ofp_header* field is used to indicate a Packet_In message. The *in_port* field indicates a current congested port, while the *buffer_len* field denotes the queue length of this congested port. The *reason* field indicates the three types of congestion messages, including *CN-L*, *CN-M*, and *CN-H*, and a recovery message, i.e., a *CR* message.

Algorithm 1 describes detailed pseudo-code for the network congestion trigger. The OF-switch is congested when current queue length is larger than *L*. Then a congestion notification message is generated and delivered to the controller. If current queue length is lower than thresholds *L* for at least 1 s, we consider that the congested network is completely recovered. Then a recovery message will be sent to the controller.

    **Algorithm 1.** Network Congestion Trigger    **Input**: packet *P* arrives **    Initial**: state = 0, type = 0, TIMER is stopped
1: CurrentLength = CurrentLength + size(*P*);2: IF (state == 0)3:   IF (CurrentLength >= *L*) // state from non-congestion state to congestion state4:   type = *CN-L*;5:   state = 1; // low congestion state6: ELSE7:   IF (state == 1 && CurrentLength >= *M* && CurrentLength < *H*)8:   type = *CN-M*9:   state = 2; // modest congestion state10:  IF (state == 2 && CurrentLength >= *H*)11:  type = *CN-H*12:  state = 3; // high congestion state13:  IF (CurrentLength < *L*)14:    IF (TIMER is stopped)15:    start TIMER for 1s;16:    IF (TIMER is expired) // congestion recovery17:    stop TIMER;18:    type = *CR*;19:    state = 0; // non-congestion state20:  Else21:  IF (TIMER exists)22:  stop TIMER;23:IF (type! = 0)24:  Message = GenerateOFMessage(type, CurrentLength);25:  sendMessage(Message); // send message to controller via OpenFlow26:  type = 0;

### 3.3. Step 2. Flow Selection

Our flow selection module is designed to maintain a flow table that contains all of TCP flow information and then categorize these flows into the background flows and burst flows. Our controller can generate a global-view flow table (GVT) that includes all of the TCP connection information in the whole network via the OpenFlow protocol.

**Figure 5:**
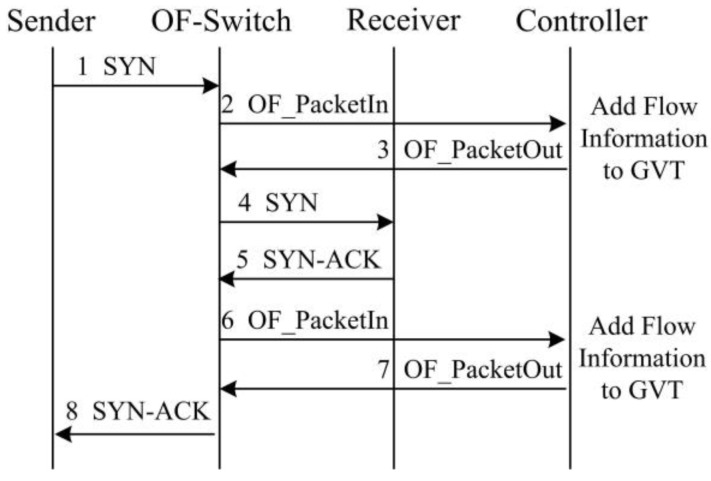
GVT entry generation with the TCP connection procedure.

**Figure 6:**
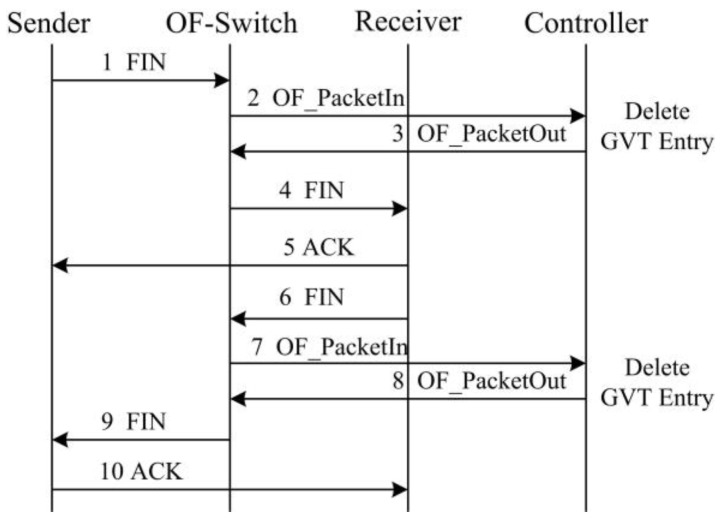
GVT entry deletion with the TCP termination procedure.

Figure 5 illustrates the procedure of recording a TCP flow information into the GVT during a new TCP establishment. Specifically, if a sender attempts to initiate a new TCP flow with a receiver, a SYN packet will be sent to the OF-switch. Since it is a new connection, there is no corresponding flow table entry at the OF-switch. Therefore, OF-switch forwards this SYN packet to the controller. The controller generates a flow table entry for this new TCP flow and records the flow information in the GVT. Then it pushes the flow table entry back to the OF-switch. According to the flow table entry, the OF-switch can forward this SYN packet to the receiver. Likewise, the controller generates a corresponding flow table entry for the SYN-ACK packet sent from the receiver and sends it to the OF-switch. Moreover, Figure 6 illustrates the GVT entry deletion in terms of the procedure of TCP connection termination. When the OF-switch receives a FIN packet and forward it to the controller, the controller will remove the corresponding TCP flow table entry and the information in the GVT.

**Figure 7:**
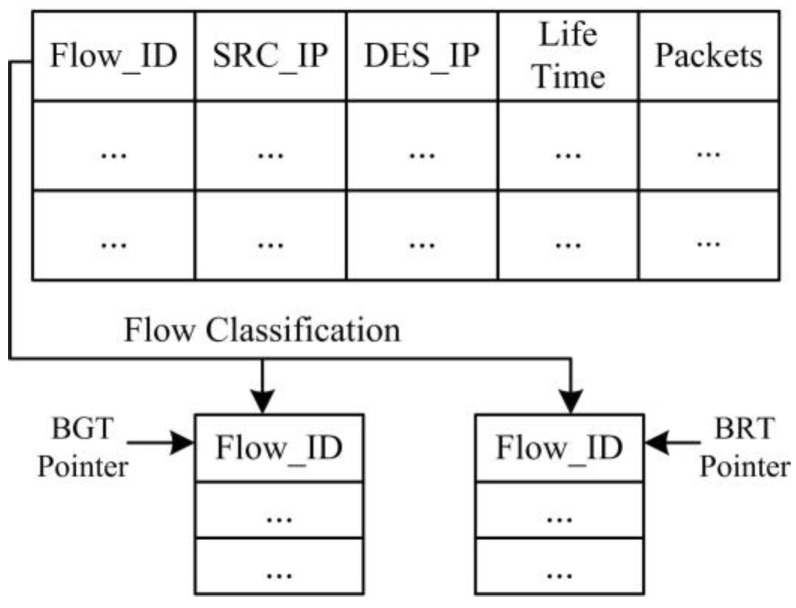
The global-view flow table.

Figure 7 depicts the format of the TCP flow recorded in a GVT. We record the lifetime of the flow and the received packets gained from statistical information at the OF-switch using OpenFlow protocol. According to the life time of the flow and the number of received packets, we can classify our flows in the GVT into background flows table (BGT) and burst flows table (BRT).

Once we derive all of the flow information in the GVT, we use a classification method to distinguish background flows and burst flows in terms of different flow traffic characteristics [3,24]. We classify the flows based on both traffic flow volume and lifetime of the flows gained from the GVT. If the traffic flow volume is larger than 1 MB and the life time is longer than 1 s, we classify this flow as a background flow and add it to the BGT as shown in Figure 7. Otherwise, if the traffic volume is smaller than 1 MB and the life time is shorter than 1 s, we classify this flow into burst flow and add it to the BRT. This classification method runs in the background at the controller. Finally, the controller can directly obtain the background flow information from BGT.

### 3.4. Step 3. Flow Rate Control

After retrieving the background flows from BGT, we estimate the send window of background flows and then discuss how to adjust the advertised window in this subsection. Suppose that *N* flows arrive at an OF-switch and then the OF-switch forwards the data of these flows to the same destination as shown in Figure 1. Assume that the round trip time (*RTT*) of these *N* flows are the same. Then we can estimate the total size of send window of each flow by:

(2) $$\sum_{i\in N}swnd_{i}\left(t\right)=C\times RTT+Q\left(t\right),$$

where $swnd_{i}\left(t\right)$ is the *swnd* of flow *i* at time *t*, *C* is the capacity of a bottleneck link, and Q(t) is the queue length at time *t*. *RTT* could be gained through a method proposed in [25], while Q(t) can be derived in the congestion notification message received from the OF-switch as shown in Figure 4. Denote *G* and *B* as the set of background flows and burst flows, respectively. *a* = |G| and *b* = |B| represent the number of background flows and burst flows, respectively, where *N = a + b*.

According to the fairness of legacy TCP, the capacity of bottleneck link will be evenly shared by each flow. Therefore, the *swnd* of each flows is given as:

(3) $$W_{swnd}=\frac{C\;\times \;RTT\;+\;Q\left(t\right)}{N},$$

When congestion happens, we reduce the bandwidth of background flows to provide burst flows with more bandwidth so as to alleviate the network congestion. To this end, we decrease *swnd* of each background flow by:

(4) $$swnd_{j}^{\prime }=\mu W_{swnd},\;\left(j\in G\right),$$

where 0<μ<1 is the given factor that implies levels of congestion.

We reduce the background flow rate of the senders by regulating the *awnd* of the corresponding TCP ACK packets from the receiver. Since we defined three congestion levels as presented in Step 1, we need to regulate the *awnd* in terms of different congestion levels. We illustrates these three cases as follows:

**Case 1.** Receiving low congestion notification (*CN-L*) message. If the controller receives a *CN-L* congestion notification message, we consider that network has entered into a low-level congestion state. Then we set $\mu =\frac{2}{3}$ in the Equation (4) and get *awnd* of each background flow by:

(5) $$awnd_{j}=\max\left(\frac{2}{3}W_{swnd},1\;\mathrm{MSS}\right),\;\left(j\in G\right),$$

where $W_{swnd}$ and $awnd_{j}$ can be calculated by Equations (3) and (5), respectively. The minimum *awnd* of background flow is 1 MSS.

**Case 2.** Receiving modest congestion notification (*CN-M*) message. If the controller receives a *CN-M* congestion notification message, we set $\mu =\frac{1}{2}$ and get the *awnd* of each background flow by:

(6) $$awnd_{j}=\max\left(\frac{1}{2}W_{swnd},1\;\mathrm{MSS}\right),\;\left(j\in G\right),$$

**Case 3.** Receiving high congestion notification (*CN-H*) message. When this situation happens, network congestion is very serious. Therefore, we use a radical remedy to set the *awnd* of the all of the flows by:

(7) $$awnd_{j}=1\;\mathrm{MSS},\;\left(j\in G\right),$$

(8) $$awnd_{m}=\max\left(\frac{C\;\times \;RTT\;+\;Q\left(t\right)\;-\;a\;\times \;MSS}{b},1\;\mathrm{MSS}\right),\;\left(m\in B\right),$$

In this case, we assign a baseline bandwidth (i.e., 1 MSS) to each background flow to reserve sufficient bandwidth for burst flows. After gaining the *awnd*, the controller generates regulation *awnd* entries and delivers them to the OF-switch.

### 3.5. Step 4. Flow Match and Regulation

To support the TCP flag-based flow table entry and *awnd* based flow regulation functionality, we revised both the controller and the Open vSwitch (version 2.3.0) to extend the existing OpenFlow protocol (version 1.3). In particular, we leverage the *oxm_type* field of the OpenFlow extensible match (OXM) in the OpenFlow protocol to define a TCP flag field so as to support the TCP flag-based flow table entry. The type of the TCP flag includes SYN, FIN, ACK, etc. Moreover, we added a new value to the existing action field in the flow table entry. The new value consists of the *awnd* regulation command, i.e., OFPT_FLOW_MOD, and the specific value of *awnd*. In this way, a new flow table entry can support the TCP flag match and flow regulation functionalities. Furthermore, we configured Open vSwitch to upgrade the priority of the flow table entries for matching the SYN and FIN packets to send these types of packets to the controller for maintaining the flow information in the GVT.

When the TCP ACK packets match the regulation *awnd* entries at the OF-switch, the *awnd* of the TCP ACK packets will be modified in terms of the value of *awnd* in the entries. In practice, the *awnd* is determined by:

(9) $$awnd_{i}^{\prime }=\min\left(awnd_{i},awnd_{r}\right),\;\left(i\in G\right),$$

where $awnd_{r}$ is the *awnd* of TCP ACK packet arriving at the OF-switch and $awnd_{i}$ is the value of *awnd* in the flow table entry for bandwidth regulation of the *i-*th background flow.

## 4. Analysis

In this section, we investigate the characteristics of the SDTCP, including control delay, flow completion time, and fairness.

### 4.1. Control Delay

We evaluate the efficiency of SDTCP by analyzing the delay introduced for controlling background flow rate. In our SDTCP mechanism, when the queue length is larger than the threshold, the OF-switch triggers a congestion notification message and regulates the *awnd* of TCP ACK packets of the corresponding background flows via the OpenFlow protocol. As a result, the regulation *awnd* entries will be generated at the controller side and transmitted to in the OF-switch. The time consumed in this procedure is referred to as the control delay.

The control delay composes of round trip transmission time between the controller and OF-switch and time of regulation *awnd* entries generation at the controller. Denote $T_{cd}$ as the control delay. Then, we have $T_{cd}=T_{bf}+T_{p}$, where $T_{bf}$ is the round trip transmission time between the controller and OF-switch and $T_{p}$ is processing time at the controller. $T_{p}$ contains the time of the flow selection and flow rate control in Step 2 and Step 3, respectively. BGT is generated in the background and the time complexity of BGT based flow selection is O(a), where *a* is the number of background flows. Moreover, the time complexity of flow rate control is O(a) as well. Since the median number of concurrent background flows is one and the 75th percentile is two [3], we can regard time complexity of processing time O(a) as O(1). Then we have $T_{cd}\approx T_{bf}$. Thus, the impact of the control delay $T_{cd}$ relies on $T_{bf}$. If the distance between the OF-switch and the controller is very close (i.e., one hop distance), and $T_{cd}$ can be virtually ignored.

### 4.2. Flow Completion Time

We theoretically analyze two cases of packet lossless and packet loss to demonstrate that the average flow completion time (FCT) can be effectively reduced by allocating more bandwidth to burst flows. We discuss the average FCT of all the flows from two aspects including packet losslessness and packet loss.

(1) Packets losslessness

Let $S_{a}$ and $S_{b}$ be the total volume of background flows and burst flows, respectively, and we assume that each flow duration is $\left[D_{s}\left(k\right),\;D_{t}\left(k\right)\right],\;$where $D_{s}\left(k\right)$ and $D_{t}\left(k\right)$ are the start time and the end time of the *k-*th flow. Therefore, the background flow duration is $\left[g_{a},h_{a}\right],$ where $g_{a}=\underset{i\in G}{min}\;D_{s}\left(i\right),h_{a}=\underset{i\in G}{max}\;D_{t}\left(i\right),$ and burst flow duration is $\left[g_{b},h_{b}\right]$, where $g_{b}=\underset{j\in B}{min}\;D_{s}\left(j\right),h_{b}=\underset{j\in B}{max}\;D_{t}\left(j\right)$. Since the background flow duration is larger than the burst flow duration, we can have $g_{a}<g_{b}<h_{b}<h_{a}$. After assigning more bandwidth to the burst flows, we assume that durations of the background flows and burst flows are $\left[g_{a}^{\prime },\;h_{a}^{\prime }\right]$ and $\left[g_{b}^{\prime },\;h_{b}^{\prime }\right]$, respectively, where $g_{a}^{\prime }=g_{a},\;g_{b}^{\prime }=g_{b}$. Then, the average sending rate of burst flows can be increased from $R_{b}$ to $R_{b}^{\prime }$, where $R_{b}^{\prime }>R_{b}$. Then we can compare the burst flow duration with SDTCP with that without SDTCP by:

(10) $$h_{b}^{\prime }-g_{b}^{\prime }=\frac{S_{b}}{R_{b}^{\prime }}<\frac{S_{b}}{R_{b}}=h_{b}-g_{b},$$

From Equation (10), we can deduce that FCT of burst flows decreases. After the background and burst flows are completed, we have:

(11) $$h_{a}^{\prime }-g_{a}^{\prime }=\frac{S_{a}+S_{b}}{C}=h_{a}-g_{a},$$

where *C* is the capacity of the network bottleneck. Thus, we can see that the FCT of background flows is the same.

As we can see from Equations (10) and (11), the average FCT of the background flows and burst flows reduces in the case of packets losslessness.

(2) Packets loss

In the packet loss case, the packet loss of the burst flows can incur the packet retransmission so as to increase the average FCT of all of the flows. When both burst flows and background flows co-exist on the same switch (or router), most of the buffer space is occupied by packets from the background flows. Thus, a small amount of buffer space can be used for the burst flows [3]. This results in a large number of packets from burst flows that are lost, leading to packet retransmission. Since the send window of burst flows is quite small, the sender may not get sufficient duplicate acknowledgements to trigger fast retransmission. Thus, the effectiveness of a TCP fast retransmit mechanism for burst flows can be substantially reduced. Then TCP retransmission timeout happens so incurring a much larger retransmission time. Therefore, TCP retransmission timeout of burst flows dominates the FCT. If we allocate more bandwidth to the burst flows, burst flows will suffer less packet loss. Finally, from the above discussion, the average FCT decreases if we assign more bandwidth to burst flows.

According to our analysis, we can reduce the FCT of burst flows using SDTCP in either the packet lossless or packet loss case, while background flows are not influenced.

### 4.3. Fairness

Since SDTCP allocates more bandwidth to burst flows, this will cause TCP unfairness between background flows and burst flows. However, this unfairness can be used to accommodate more burst flows for alleviating the TCP incast problem [24] and reduce transmission time of deadline-aware burst flows [26]. We observe that our bandwidth allocation of SDTCP is weighted proportional fairness that we prove in Proposition 1. According to the SDTCP mechanism, both the bandwidth allocation among the background flows and that among burst flows are fair.

**Proposition** **1.** In our SDTCP, the bandwidth allocation of TCP flows is weighted proportional fairness.

**Proof.**  Denote$\;\left\{x_{r}\right\}$ and $W_{r}$ (r∈R) as a set of TCP flow rates and the weight of the TCP flow, respectively, where R is the set of all TCP flows. According to [27],$\;\left\{x_{r}\right\}$ satisfies weighted proportional fairness if only if for any other TCP flow rates, e.g., $\left\{y_{r}\right\}$, the sum of weighted proportional changes is zero or negative, i.e., $\underset{r\in R}{\sum }W_{r}\frac{y_{r}-x_{r}}{x_{r}}\leq 0$. According to our SDTCP mechanism, assume that the rate and the weight of the flow $x_{i}\;$is $\frac{\mu W_{swnd}}{RTT}$ and $\frac{\mu }{N}$, where i∈G and G is the set of background flows. We also assume that the rate and the weight of the flow $x_{j}\;$is $\frac{\frac{N-au}{b}W_{swnd}}{RTT}$ and $\frac{1-a\frac{\mu }{N}}{b}$, where j∈B and B is the set of burst flows. For simplicity, we let $R_{base}=\frac{W_{swnd}}{RTT}$. For any other flow rates set, i.e., $\left\{y_{r}\right\}$, we let $y_{i}=\gamma R_{base}$ for i∈G and $y_{j}=\frac{N-a\gamma }{b}R_{base}$ for j∈B. Hence, we obtain:

$$\underset{r\in R}{\sum }W_{r}\frac{y_{r}-x_{r}}{x_{r}}=\underset{r\in R}{\sum }W_{r}\frac{y_{r}}{x_{r}}-\underset{r\in R}{\sum }W_{r}=\underset{i\in G}{\sum }W_{r}\frac{y_{i}}{x_{i}}+\underset{j\in B}{\sum }W_{r}\frac{y_{j}}{x_{j}}-1\;=\underset{i\in G}{\sum }\frac{\mu }{N}\frac{\gamma R_{base}}{\mu R_{base}}+\underset{j\in B}{\sum }\frac{1-a\frac{\mu }{N}}{b}\frac{\frac{N-a\gamma }{b}R_{base}}{\frac{N-a\mu }{b}R_{base}}-1=\underset{i\in G}{\sum }\frac{\mu }{N}\frac{\gamma }{\mu }+\underset{j\in B}{\sum }\frac{1-a\frac{\mu }{N}}{b}\frac{N-a\gamma }{N-a\mu }-1\;=a\frac{\gamma }{N}+\frac{N-a\gamma }{N}-1=0\leq 0$$

Therefore, the bandwidth allocation of SDTCP is weighted proportional fairness.

### 4.4. Scalability

Our SDTCP mechanism is a centralized approach. The controller has a global visibility of the whole network and is responsible for all switches in the DCN. As a result, the controller in our SDN architecture is prone to suffer from scalability issues due to its centralized nature. According to [7,28], the ability of a central controller can be scaled to 20 million flows per second through parallelism (i.e., use multiple CPUs per controller and multiple controllers). Moreover, the system performance and control delay are evaluated in Section 5.2.5 and the experimental results show that our controller can process 1500 concurrent flows within about 100 μs. Therefore, a centralized controller method is practical in data centers.

## 5. Experimental Results

In this section, we present the experimental evaluation of SDTCP in a DCN environment. We compare SDTCP with TCP New Reno, DCTCP, and SAB. The reason of choosing these three protocols is that TCP New Reno is widely used in practice, DCTCP is the most popular data center transport protocol, and SAB is similar to SDTCP.

### 5.1. Experimental Setup

We implement the SDTCP mechanism in Mininet (version 2.2.1) [29], which uses Open vSwitch (version 2.3.0) as the OF-switch. Mininet is installed in a server where the hardware profile includes 2.4 GHz Intel CPUs with eight cores, 16 GB RAM, and a 1 T hard disk, and the operating system is Ubuntu 14.04.2 with Linux kernel version 3.16. In addition, our SDTCP controller is implemented on top of the Floodlight [30] and is deployed in a laptop with a 1.9 GHz Intel I5 Core, 4 GB RAM, and a 500 GB hard disk, and the operating system is also Ubuntu 14.04.2.

For DCTCP implementation, we use public code from [31] and add ECN capability to SYN packets [32]. Meanwhile, we use TCP New Reno [33] (named TCP for short in the later experiments) as our congestion control algorithm and disable the delayed ACK mechanism. In our experiments, we implement the SAB with a fixed value for MSS and ε=0.5 where ε is the proportion of available buffer space.

**Figure 8:**
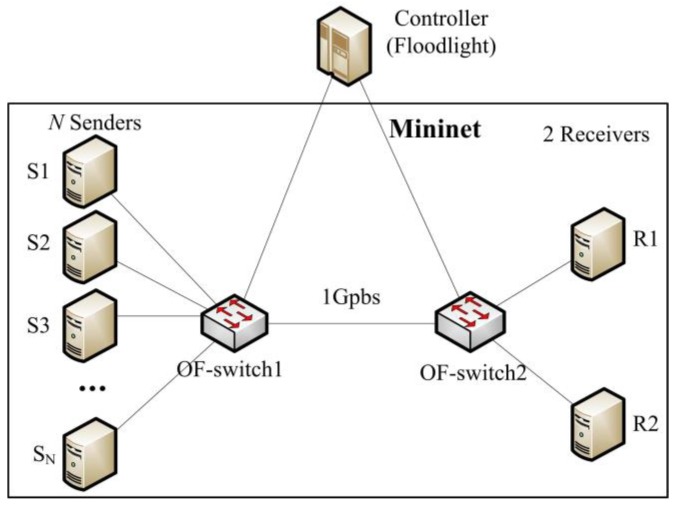
Experiment topology.

The experimental network topology adopts the typical TCP incast scenario as shown in Figure 8, where *N* senders are connected to the OF-switch1, two receivers are connected to the OF-switch2 and a link between OF-switch1 and OF-switch2. The links in our network topology have a 1 Gbps throughput and a latency of 33 μs each to create about 200 μs fixed RTT. We can know that TCP incast congestion happens in the intermediate link between OF-switch1 and OF-switch2.

Given the poor TCP performance with 200 ms minimum RTO, we use a minimum RTO of 30 ms for the TCP retransmission timeout. Assume MSS is 1460 byte. We allocate a static buffer size of 100 packets (100 × 1.5 KB = 150 KB) to each OF-switch port. For DCTCP, we set the ECN marking threshold in the switch to be 20 packets (20 × 1.5 KB = 30 KB). For our SDTCP, we set the threshold *L*, *M* and *H* to be 30 packets, 60 packets, and 85 packets respectively.

### 5.2. Experimental Results

#### 5.2.1. SDTCP Goodput

(1) SDTCP goodput with different concurrent flows

TCP incast usually involves a large number of concurrent flows. In this experiment, we examine the goodput of SDTCP, DCTCP, TCP, and SAB under different *N*, the number of concurrent flows. We set up the following experiment scenario: there are *N* flows competing for a 1 Gbps link, and one of them is the background flow with infinite data and the others are burst flows. We fix the flow size generated by each flow and the data amount of all of the flows is increased with the number of flows is increasing. Given the poor performance of the lower flow size, we set each flow size to be 512 KB.

**Figure 9:**
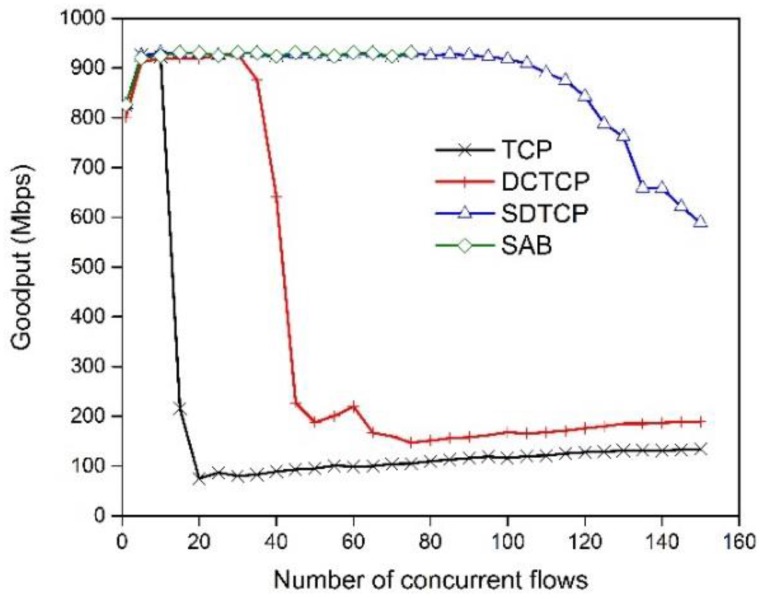
Goodput of SDTCP, TCP, DCTCP, and SAB.

Figure 9 shows the goodput of SDTCP, TCP, DCTCP, and SAB as we vary the number of concurrent flows from 1 to 150. As shown in this figure, the goodput of TCP collapses when the number of flows is larger than about 10. This phenomenon of goodput collapsing the DCTCP happens when the concurrent number of flows reaches 37. Both SDTCP and SAB performs well until the number of flows reaches 70. The link utilization is about 92%. When the number of concurrent flows is larger than 70, our experiment for SAB cannot be executed because our system runs out of resources resulting in system crash. This indicates that SAB needs much more system resources than SDTCP because that the mechanism of SAB needs to modify every packet that passes through the switch. If the number of concurrent flows continues to increase until 100, the goodput of SDTCP is slightly decreased. This is because that queue length will reach threshold H when 100 flows send packets synchronously with an initial window of TCP, i.e., 1 MSS. SDTCP will consume more time (three congestion notification messages) to assign bandwidth fairly to each TCP flow, and so may slightly degrade network goodput. Finally, when the number of concurrent flows reaches 120, SDTCP experiences goodput degradation because of some packets loss. In this experiment, SDTCP can easily handle 100 concurrent flows and significantly improve the network performance over the incast scenario.

**Figure 10:**
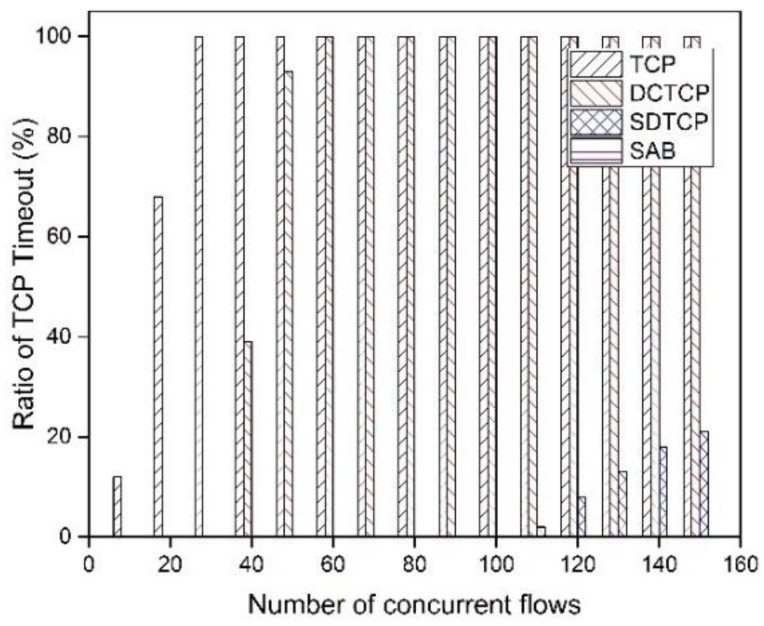
Ratio of timeout with fixed flow size.

With the same experiment, we present the timeout ratio for different protocols. The timeout ratio is the ratio between the number of flows that experience at least one timeout and that of all of flows. From the Figure 10, we observe that TCP and DCTCP experience 100% of the timeout ratio when the number of concurrent flows reaches 30 and 60, respectively. The highest timeout ratio for SDTCP is about 20% when the number of concurrent flows reaches 150. The reason for SDTCP to effectively reduce the timeout ratio is that SDTCP reduces senders’ *swnd* of the background flow to relieve the increment of the queue length. With the same reason discussed above, we cannot gain the timeout ratio for SAB when the number of concurrent flows is larger than 70.

(2) SDTCP goodput with consecutive time

To show the detail of goodput of background flows and burst flows, we consider the scenario that one background flow with infinite data size transmits at first and after 11 s, 10 burst flows with 512 KB data start to transmit synchronously.

**Figure 11:**
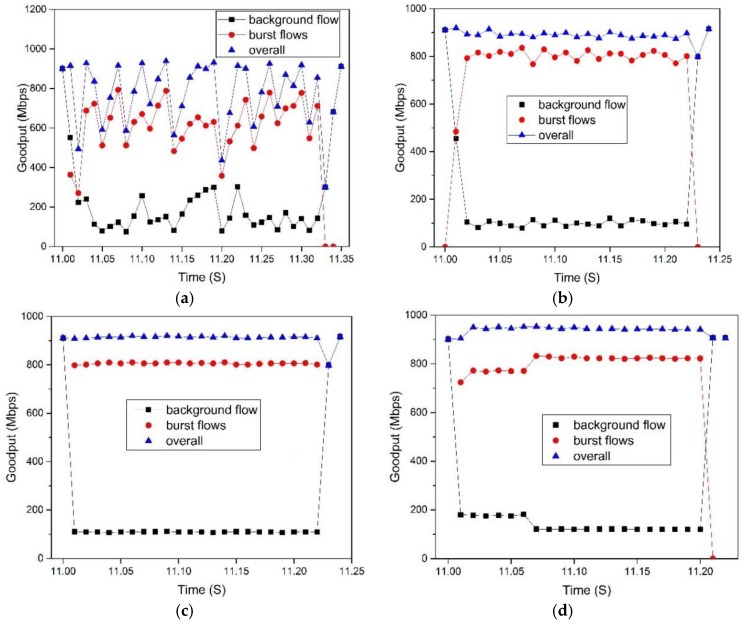
Goodput of background flows and burst flows with 512 KB data: (**a**) TCP; (**b**) DCTCP; (**c**) SAB; and (**d**) SDTCP.

Figure 11, we show the comparison results of TCP, DCTCP, SDTCP, and SAB. We observe that before burst flows arrive, background flow can achieve the steady goodput of about 910 Mbps. However, TCP experiences goodput collapse when concurrent burst flows are injected into the network, which leads to TCP incast. Both the background and burst flows suffer poor performance, as shown in the Figure 11a. DCTCP, SAB, and SDTCP do not suffer any TCP incast problems when the number of concurrent flows are 10. However, the goodput curve of TCP and DCTCP varies up and down, while that of SAB and SDTCP remains stable. This is because both SAB and SDTCP regulate the sending rate. In the Figure 11d, SDTCP reduces the bandwidth of background flow to assign more bandwidth to burst flows and, hence, the goodput of burst flows for SDTCP is larger than that of DCTCP and SAB. This leads to the shorter transmission time of SDTCP which can be seen from the results of comparing TCP, DCTCP, and SAB.

#### 5.2.2. OF-Switch Queue Length

In this experiment, we evaluate the effectiveness of SDTCP on the switch buffer. To this end, the buffer length is logged during the experiment. We start 20 burst flows and 50 burst flows at time 10, respectively, and each flow size is 500 KB.

**Figure 12:**
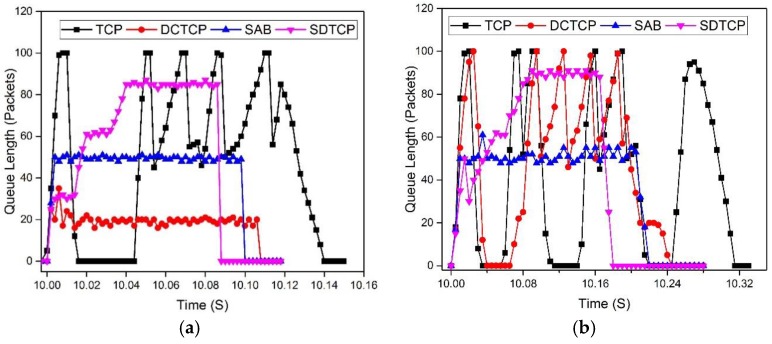
Queue length dynamics with 500 KB flow size: (**a**) 20 burst flows; and (**b**) 50 burst flows.

The result is shown in the Figure 12. Before time 10 s, the queue is empty in both situations. In the situation of 20 burst flows, the TCP suffers one retransmission timeout, several buffer overflows, and causes wide oscillations as shown in the Figure 12a. DCTCP and SAB maintain a stable queue length while DCTCP occupies a smaller buffer. The buffer length of SDTCP increases with the varying time, and finally occupies an 85 packet queue length. As a result, SDTCP will complete its transmission first.

In Figure 12b, we show the queue length with 50 burst flows. In this case, both TCP and DCTCP reach maximum queue length which leads to packet loss and timeout. However, TCP suffers three retransmission timeouts and DCTCP experiences one retransmission timeout. The queue length of SAB and SDTCP is similar to that of 20 burst flows.

#### 5.2.3. Flow Completion Time

In DCN, the flow completion time is an important metric for application. In this experiment, we fix our flow size to 100 KB and compare TCP, DCTCP, SAB, and SDTCP to gain the FCT of burst flows with different numbers of concurrent flows.

**Figure 13:**
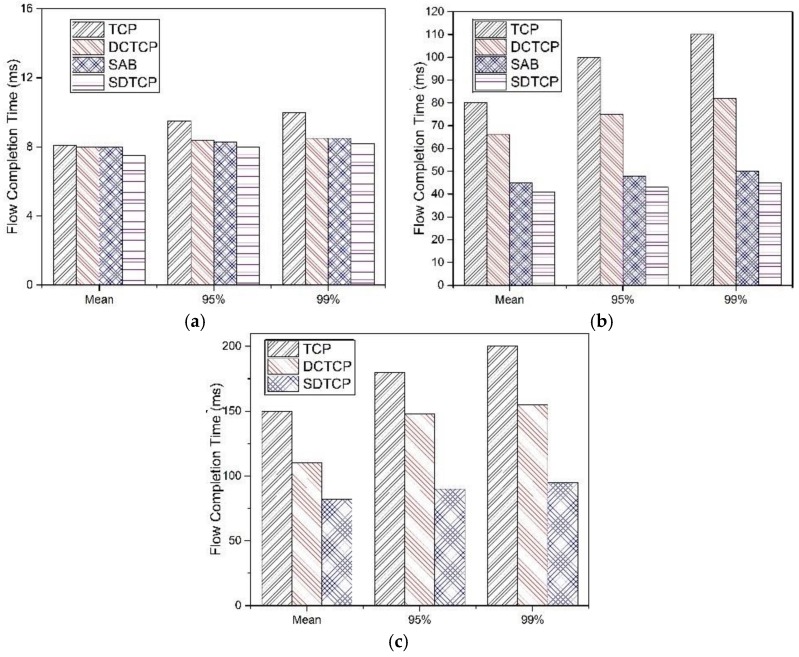
Mean, 95th, and 99th percentile FCT with different concurrent number of flows: (**a**) *N* = 10; (**b**) *N* = 50; and (**c**) *N* = 100.

Figure 13 shows the mean, 95th percentile and 99th percentile of the FCT for different numbers of burst flows. We observe that SDTCP performs the best of these protocols, especially at *N* = 100. This is because burst flows in SDTCP can get more bandwidth than the other three protocols, which results in less packet loss. In the case of *N* = 10, we find that no TCP timeout happens, while TCP suffers some packets retransmission. In the case of *N* = 50 and *N* = 100, both TCP and DCTCP suffer several TCP timeouts and, therefore, the FCT of TCP and DCTCP are much longer than that of SAB and SDTCP.

**Figure 14:**
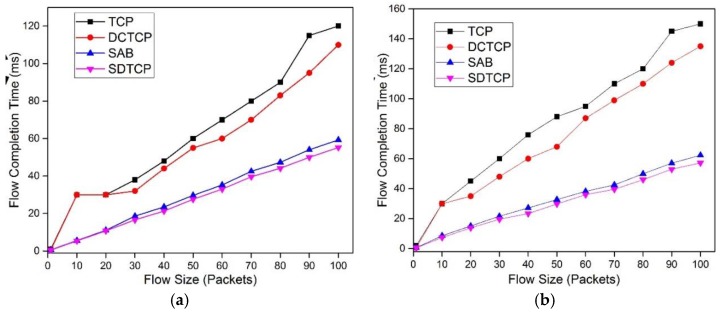
Mean and 99th percentile FCT for 50 burst flows: (**a**) m FCT; and (**b**) 99th percentile FCT.

Figure 14 shows the FCT of burst flows with flow size increasing from one packet to 100 packets. We observe that when flow size is 30 packets, the mean FCT of TCP and DCTCP is 38 ms and 32 ms, respectively, indicating timeout happens at least once in the experiments, while the 99th percentile FCT of TCP and DCTCP can reach 60 ms and 55 ms, respectively. With the flow size increasing, the FCT of four protocols increases. However, the FCT of TCP and DCTCP is much larger than that of SAB and SDTCP. The reason is that TCP and DCTCP suffer many more TCP timeouts. For both the mean and 99th percentile of FCT, we can see the advantage of SDTCP over SAB, although the difference is slight.

#### 5.2.4. Fairness

**Figure 15:**
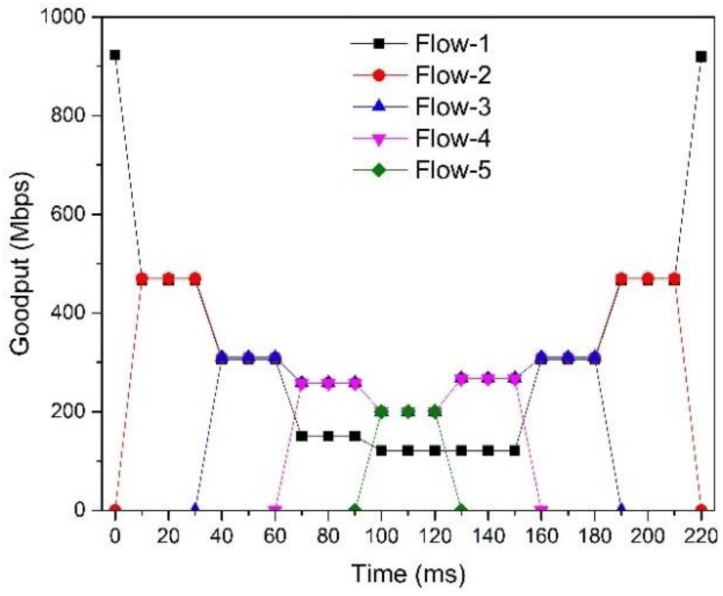
SDTCP fairness.

In this experiment, we experiment with a scenario with one background flow (Flow-1) and four4 burst flows that start and stop each in a predetermined order to test the fairness. In Figure 15, we can observe that the goodput of SDTCP is close to the maximum of the link capacity. When congestion does not happen (before 60 ms), the flows of SDTCP achieve their fair goodput. However, after congestion happens, the background flow, i.e., Flow-1, gives their bandwidth to burst flows and, therefore, burst flows achieve higher goodput. As we discussed in Section 4.3, SDTCP satisfies weighted proportionally fairness.

#### 5.2.5. Controller Stress Test

In our SDTCP mechanism, the flow’s first packet arriving at the OF-switch is forwarded to the controller through the Packet_In messages so as to determine the flow table and form the GVT. Therefore, when the number of concurrent flows is large, the system performance of the controller, including the CPU, memory, and bandwidth, may be an issue. In order to ensure the scalability and feasibility of the SDTCP, the controller must withstand the stress test.

**Figure 16:**
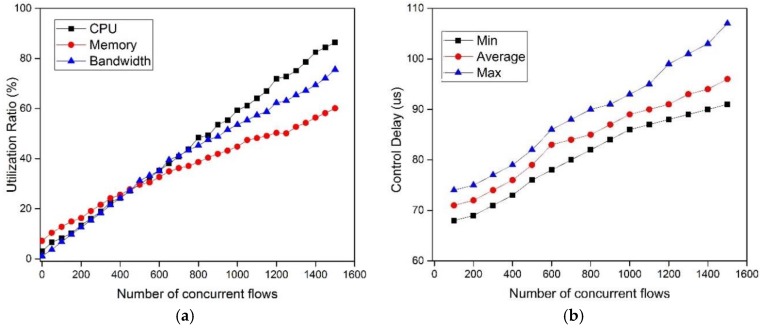
The utilization and control delay with different numbers of flows: (**a**) The utilization of system performance; and (**b**) the control delay.

In this scenario, the experiment topology is built as in Figure 8. We record the utilization of system performance, including the CPU, memory, bandwidth, and control delay while the number of concurrent flows varies from one to 1500, which is a reasonable value in DCN [2,3]. As shown in the Figure 16a, when the number of flows increases, the utilization ratio of CPU, memory, and bandwidth, is increasing linearly. We know that the more concurrent flows, the more resources are consumed by the controller to handle the Packet_In messages.

The control delay is one of the important metrics for our SDTCP, as we discussed in Section 4.1. In this experiment, we evaluate the control delay with the number of GVT entries increasing from 100 to 1500, in which one percent of the total entries are background flow entries. This control delay contains the 66 μs basic transmission delay (two links including 66 μs delay) and the controller processing time. The result is shown in Figure 16b. The average control delay varies between 70 μs and 95 μs, which is much smaller than RTT. This is because background flows can be selected directly in the BGT, which is generated in the background. In summary, the result shows that control delay of the SDTCP should not affect system performance. However, the better system capabilities, the better performance we can obtain.

## 6. Conclusions

In this paper, we present SDTCP, a novel transport protocol for providing high-throughput transmission service for IoT applications. When burst flows arrive at the switch and queue length is larger than the threshold, SDTCP reduces the sending rate of background flows proactively to guarantee burst flows by adjusting the advertised window of TCP ACK packets of the corresponding background flows. SDTCP needs no modification to the existing TCP stack and makes use of an extended OpenFlow protocol, a technology already available in current commodity switches. We evaluate SDTCP through extensive experiments. Our results demonstrate that the SDTCP mechanism guarantees high throughput for burst flows effectively without starving background flows. Moreover, the FCT of SDTCP is much better than other protocols. Therefore, SDTCP can deal with the TCP incast problem excellently.
